# Living on the edge: comparative phylogeography and phylogenetics of *Oreohelix* land snails at their range edge in Western Canada

**DOI:** 10.1186/s12862-019-1566-1

**Published:** 2020-01-06

**Authors:** Z. W. Dempsey, C. P. Goater, T. M. Burg

**Affiliations:** Department of Biological Sciences, University of Lethbridge, 4401 University Drive, Lethbridge, AB T1K 3M4 Canada

**Keywords:** Glaciation, Isolation, Secondary Contact, Sky Islands, Speciation, Hybridization

## Abstract

**Background:**

The biodiversity and distributions of terrestrial snails at local and regional scales are influenced by their low vagility and microhabitat specificity. The accessibility of large-bodied species and their characteristically high levels of genetic polymorphism make them excellent ecological and evolutionary models for studies on the phylogeography, phylogenetics, and conservation of organisms in fragmented populations. This study aims to elucidate the biodiversity, systematics, and distributions of genetic lineages within the genus *Oreohelix* at the northern and western periphery of their range.

**Results:**

We found four mitochondrial clades, three of which are putative subspecies of *Oreohelix subrudis*. One clade was geographically widespread, occurring within numerous sites in Cypress Hills and in the Rocky Mountains, a second was geographically restricted to the Rocky Mountains in Alberta, and a third was restricted to the Cypress Hills region. A fourth clade was the small-bodied species, *O. cooperi*. ITS2 sequence and screening data revealed three genetic clusters, of which one was *O. cooperi*. Cluster 1 contained most individuals in COI clade X and some from clade B and cluster 2 was predominantly made up of individuals from COI clades B and B′ and a few from clade X. ITS2 alleles were shared in a narrow contact zone between two COI clades, suggestive of hybridization between the two.

**Conclusions:**

A sky island known as Cypress Hills, in southeastern Alberta, Canada, is a biodiversity hotspot for terrestrial land snails in the genus *Oreohelix*. The observed phylogeographic patterns likely reflect reproductive isolation during the Last Glacial Maximum, followed by secondary contact due to passive, long-range dispersal resulting from low vagility, local adaptation, and complex glacial history.

## Background

The distribution of species is determined by their contemporary and historical responses to localized conditions within a heterogeneous landscape, biological characteristics such as body size and life history, and vagility [1]. Species distributions are dynamic as is the habitat in which they are found. Species ranges undergo large fluctuations over time, particularly during the Pleistocene due to a series of glaciations and climate change [2]. Ultimately, a species spatial distribution within a landscape determines population connectivity and is critical for population persistence and for maintaining genetic diversity. Typically, peripheral populations experience lower gene flow, reduced genetic diversity, and tend to occur in marginal habitats [3]. Because core and peripheral populations are often locally-adapted to different environments, selection can occur and reduce gene flow between them as immigrants and any of their offspring will tend to have reduced fitness. In some cases, these marginal populations can become completely isolated from the core populations and give rise to new species [4]. Population connectivity is not the only challenge, population dynamics are also important. Peripheral population sizes are often small and survival is dependent on maintaining a critical population size to prevent the negative effects of genetic drift or demographic stochasticity. The impact of small population size is more pronounced in peripheral populations, especially in species with limited dispersal ability.

Species with low vagility, such as terrestrial gastropods, lack a reliable method of long-distance dispersal, and can persist in isolated, disconnected populations. Indeed, some land snails can limit their life-time home ranges to only a few meters [5]. As a result, land snails are often severely restricted in their ability to respond to rapid and localized anthropogenic changes such as habitat alteration, fragmentation, introduced species, and climate change [6]. These traits make land snails excellent models for conservation biologists seeking to understand and mitigate biodiversity loss and they also make them excellent models for phylogeographic and phylogenetic studies seeking to understand the roles of history and selection in the origin and maintenance of genetic diversity [5, 7]. Studies completed over the past two decades document unusually high intraspecific sequence variation in mitochondrial DNA in some terrestrial snails [8] providing an additional opportunity to understand how natural selection and historical factors have shaped their current distributions [9, 10]. Since land snails often exist within peripheral, unconnected populations that experience reduced gene flow, the importance of biogeographic and phylogenetic differences between core and peripheral populations can be straightforward to assess. Since extensive population fragmentation and limited dispersal also lead to conservation concerns, it is best to examine a range of genetic markers. For example, incorporating nuclear markers provides for a broader context than using a single, maternally inherited marker and can yield important differences [10, 11].

Terrestrial ‘mountain snails’ in the genus *Oreohelix* (Stylommatophora; Oreohelicidae) are large-bodied (up to 2 cm in shell diameter), air-breathing, and conspicuous components of the terrestrial mollusc community in high-elevation habitats in western North America [12, 13]. The 80 described species within the genus [13] are found in a broad range of habitats that extend north-south from Canada to northern Mexico, and east-west from the Black Hills in South Dakota to the Sierra Nevada Mountains in California. Within this continent-wide distribution, individual species vary extensively in the breadth of their habitat requirements and in their patterns of endemicity. Whereas species such as *O. subrudis* and *O. strigosa* occur throughout much of the range of the genus, others are described from single limestone sky islands, mountaintops, or isolated mountain valleys [12]. Patterns of strong endemicity and concerns regarding declines in local population sizes have led to conservation concerns. Currently, of the 19 species of *Oreohelix* considered to occur in Montana, 42% are listed as ‘Species of Concern’ [14] and one of three Oreohelids in Wyoming is listed as vulnerable.

Oreohelid mountain snails form an important component of invertebrate communities, particularly in the Rocky Mountains, Intermountain West, and other sky islands of western North America. The northern part of their range extends into southwestern Canada, a region heavily impacted during the last glacial maximum [15, 16]. Cypress Hills Interprovincial Park (Cypress Hills) is an elevated plateau approximately 400 km^2^ in southeastern Alberta and southwestern Saskatchewan surrounded by prairie and farmland. The plateau remained an unglaciated nunatak during the last glacial maximum [16] and is separated from the Rocky Mountains to the west by approximately 250 km. Despite the distance, the slopes of Cypress Hills contain many of the same flora and fauna characteristic of the Canadian Rocky Mountains [17]. While sky islands in the Intermountain West were once connected by habitat corridors that land snails were capable of traversing [12], the Cypress Hills and adjacent sky islands to the south in Montana have no history of connectivity.

The biodiversity, systematics, and distributions of Oreohelid mountain snails located at the northern and western range limits in western Canada are poorly known. In a recent study, we utilized molecular markers and traditional morphological assessments to show that the mountain snail, *O. cooperi*, previously considered to be endemic to the Black Hills region of South Dakota and Wyoming, was present in isolated, high-elevation sites in Cypress Hills Park in southern Alberta [18]. The results of that study further indicated that *O. cooperi* was sympatric with at least two other Oreohelid snails within the Cypress Hills sky island. The systematics, biodiversity and distributions of the Oreohelids in this region, and in Canada in general, are unknown. More generally, phylogenetic relationships between Oreohelid lineages present in this sky island relative to those found in the Rocky Mountains to the west and to the south in the U.S. and Mexico are unknown.

The objective of this study is to characterize phylogeographic and phylogenetic patterns for *Oreohelix* spp. that occur at sites located along their western and northern range edge. One focus is to characterize regional and local phylogenetic patterns for Oreohelids in the Cypress Hills sky island. A second focus is to utilize the results of previous phylogenetic studies involving Oreohelids sampled further south and west [12, 19] to evaluate the role of past glaciation and dispersal in determining the contemporary distributions of *Oreohelix* spp. in western Canada.

## Results

### COI sequences

**Fig. 1:**
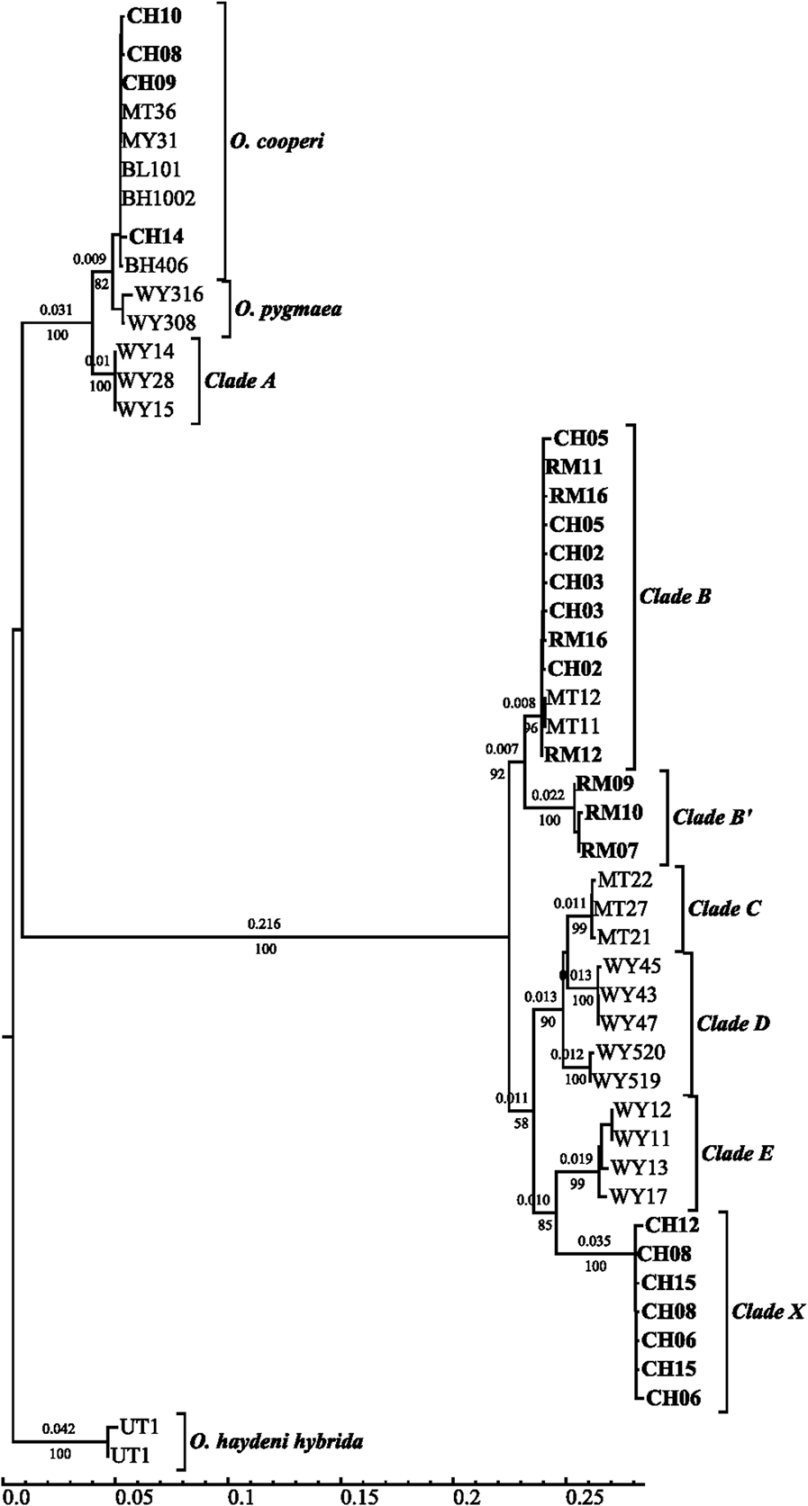
Phylogenetic tree of COI haplotype data including sequences from [10]. Branch lengths and scale are proportional to average substitutions per site above branches. Bootstraps values below nodes are based on 1000 replicates

COI sequencing of 247 individuals revealed four genetically distinct and well-supported mitochondrial clades (B, B′, X and *O. cooperi*, Fig. 1, accession numbers MN695417-MN695663). Automated selection of a phylogenetic tree model based on AICc showed the best score (−lnL = 1456) for a maximum likelihood model: Kimura 81 with gamma distribution (K81 + G; gamma shape of 0.1882; Fig. 1). The Kimura model was used to estimate sequence divergence, and missing data were analyzed with pairwise deletion setting. Sequence divergence between clades ranged from 3 to 26% with an average clade divergence of 15.2% ± 9.9%, while average sequence divergence within clades was less than 2%.

**Fig. 2:**
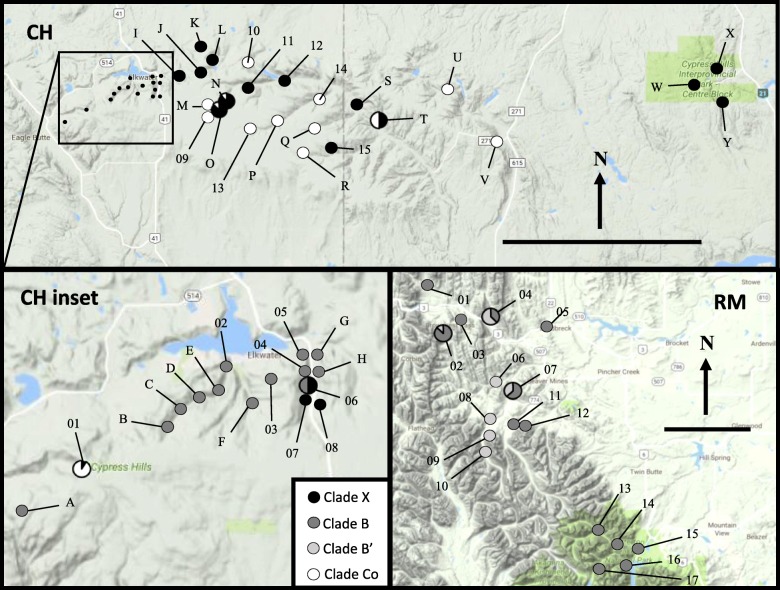
Relief image of *Oreohelix* COI sequencing (numbers) and screening (letters) results in Cypress Hills (CH) and the Rocky Mountains (RM). Detailed information on sample sizes and mtDNA clades are in Table 1. The scale bar in each inset represents 20 km. Map Data: Google Earth, CNES/Airbus, Landsat/Copernicus, Maxar Technologies (2018). *Oreohelix subrudis* clades are represented by B, B′, and X. *Oreohelix cooperi* is represented by Co

In samples collected from the Cypress Hills, all of the snails found on shrub-dominated slopes clustered into a single clade that matched *O. cooperi* mitochondrial DNA from [12] and [18]. The snails from habitats adjacent to Elkwater Lake in the western side of Cypress Hills clustered into another clade that had 99.8% sequence similarity to *Oreohelix sp. B* [12], henceforth referred to as Clade B (Fig. 1). The remainder of snails found throughout eastern Cypress Hills clustered into a third clade with 96% sequence similarity with *Oreohelix sp. E* [12], hereafter referred to as Clade X. At sites adjacent to Highway 41 (CH04–07), there was a sharp gradient of mitochondrial groups, where a distance of less than 400 m separated Clades B and X (Fig. 2). In that region, CH06 was the only site that contained both Clade B and X snails, whereas all the sites adjacent to CH06 contained a single mitochondrial clade (Figs. 2). There were four instances when *O. cooperi* was found with another COI Clade at the same site in Cypress Hills (CH01, CHO, CHN, and CHT), indicating that these species can exist in sympatry in ecotones in Cypress Hills.

For the snails collected from the Rocky Mountains, two sister clades were identified: Clades B and B′. Clade B′ snails were confined to seven sites within a small geographic area in the Castle area and Crowsnest Pass (RM02, 04, 06–10). At four of those sites, all of the snails sampled contained B′ mitochondrial DNA (Fig. 2). Overall, the Rocky Mountains snails had moderate genetic diversity (h = 0.54 compared to 0.75 in CH, and π = 0.0128 compared to 0.0805 in CH) at the COI locus, and low genetic diversity within sites (Table 1). If COI sequences from *O. cooperi* are removed from the analyses, CH snails had h = 0.63 ± 0.03 and π = 0.0278 ± 0.0006, which is still higher than the Rocky Mountains. Individually, each clade in Cypress Hills had a lower haplotype diversity than Cypress Hills as a whole (Clade B h = 0.17 ± 0.05, Clade X h = 0.33 ± 0.09). Only the CH04 site consisted of a single haplotype, whereas six Rocky Mountains sites consisted of a single haplotype (Table 1).

**Table 1:** Summary of genetic polymorphism statistics for COI sequence data for *Oreohelix* spp. analyzed by site, clade, and region including number of individuals (n), haplotypes (Hap), number of variable sites (V sites), and haplotype (h) and nucleotide (π) diversities. Site locations are described in Fig. 4. *Oreohelix subrudis* clades are represented by B, B′, and X. *Oreohelix cooperi* is represented by Co

Site	Clade	n	Hap	V sites	h ± SD	π ± SD
CH01	B/Co	11	2	95	0.18 ± 0.14	0.0282 ± 0.0223
CH02	B	15	3	2	0.26 ± 0.14	0.0004 ± 0.0003
CH03	B	8	3	2	0.46 ± 0.20	0.0008 ± 0.0004
CH04	B	9	1	0	0.00 ± 0.00	0.0000 ± 0.0000
CH05	B	7	3	2	0.52 ± 0.21	0.0010 ± 0.0005
CH06	X/B	8	7	38	0.96 ± 0.08	0.0306 ± 0.0064
CH07	X	8	3	2	0.68 ± 0.12	0.0013 ± 0.0003
CH08	X	12	2	1	0.17 ± 0.13	0.0003 ± 0.0002
CH09	Co	7	2	1	0.29 ± 0.20	0.0005 ± 0.0004
CH10	Co	8	4	4	0.79 ± 0.11	0.0026 ± 0.0005
CH11	X	10	2	1	0.20 ± 0.15	0.0003 ± 0.0003
CH12	X	5	2	2	0.40 ± 0.24	0.0014 ± 0.0008
CH13	Co	8	4	2	0.75 ± 0.14	0.0015 ± 0.0004
CH14	Co	8	7	6	0.96 ± 0.08	0.0032 ± 0.0007
CH15	X	5	3	3	0.70 ± 0.22	0.0020 ± 0.0008
RM01	B	8	2	1	0.25 ± 0.18	0.0004 ± 0.0003
RM02	B/B′	8	3	19	0.68 ± 0.12	0.0078 ± 0.0050
RM03	B	7	1	0	0.00 ± 0.00	0.0000 ± 0.0000
RM04	B/B′	8	4	19	0.75 ± 0.14	0.0157 ± 0.0035
RM05	B	8	2	1	0.54 ± 0.12	0.0008 ± 0.0002
RM06	B′	8	1	0	0.00 ± 0.00	0.0000 ± 0.0000
RM07	B/B′	7	6	22	0.95 ± 0.10	0.0187 ± 0.0035
RM08	B′	8	2	1	0.43 ± 0.17	0.0007 ± 0.0003
RM09	B′	7	3	2	0.67 ± 0.16	0.0012 ± 0.0004
RM10	B′	8	3	2	0.68 ± 0.12	0.0013 ± 0.0003
RM11	B	7	2	1	0.29 ± 0.20	0.0004 ± 0.0003
RM12	B	7	3	2	0.67 ± 0.16	0.0012 ± 0.0004
RM13	B	4	1	0	0.00 ± 0.00	0.0000 ± 0.0000
RM14	B	3	1	0	0.00 ± 0.00	0.0000 ± 0.0000
RM15	B	8	1	0	0.00 ± 0.00	0.0000 ± 0.0000
RM16	B	8	3	2	0.46 ± 0.20	0.0008 ± 0.0004
RM17	B	4	1	0	0.00 ± 0.00	0.0000 ± 0.0000
CH	B/Co/X	129	19	102	0.75 ± 0.02	0.0805 ± 0.0032
RM	B/B′	118	9	20	0.54 ± 0.04	0.0128 ± 0.0008
*O. cooperi*	Co	41	7	7	0.27 ± 0.09	0.0006 ± 0.0002
*O. sp. B*	B	121	10	10	0.17 ± 0.05	0.0004 ± 0.0001
*O. sp. B′*	B′	40	9	7	0.59 ± 0.09	0.0014 ± 0.0003
*O. sp. X*	X	45	9	11	0.33 ± 0.09	0.0008 ± 0.0003
Total	B/B′/Co/X	247	25	108	0.74 ± 0.02	0.0598 ± 0.0040

### ITS2 sequences

**Fig. 3:**
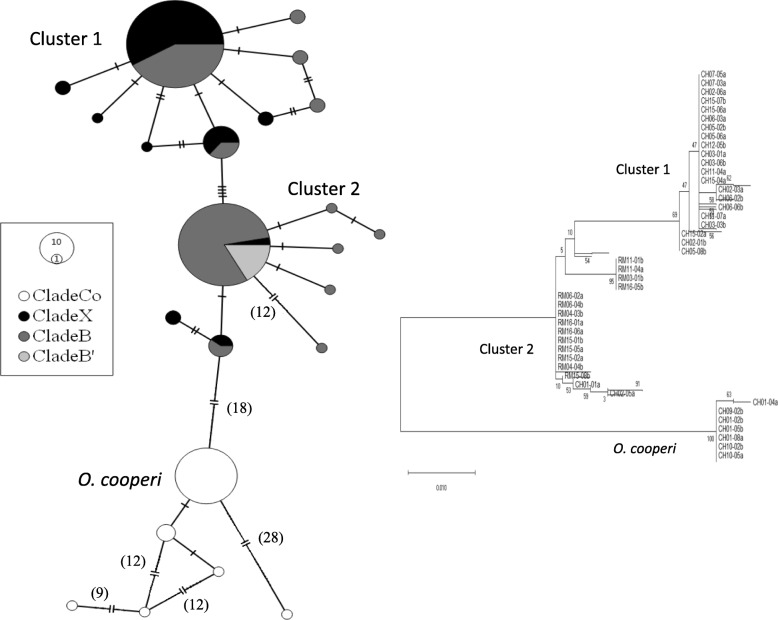
Minimum spanning network constructed for the ITS2 locus. Dashes and numbers in brackets represent number of base pair differences between alleles. Size of the circle is proportional to the number of individuals sharing that allele. Shading corresponds to mitochondrial clade. *Oreohelix subrudis* clades are represented by B, B′, and X. *Oreohelix cooperi* is represented by Co

Maximum likelihood analysis of ITS2 sequence data revealed three distinct clusters including one matching the COI *O. cooperi* clade (Fig. 3, accession numbers MN695664-MN695891). The most commonly sampled cluster (Cluster 1) was populated predominantly by COI Clade X snails, while the next most populous cluster (Cluster 2) was dominated primarily by COI Clades B and B′ (Table 2). ITS2 alleles were shared between COI Clades X, B, and B′, but no alleles were shared with *O. cooperi*. Mitochondrial Clade X contained only 11 individuals that had alleles from the Clade B ITS2 cluster, each of which was found close to the contact zone for B and X (Table 2). Forty four of the Clade B and X snails in Cypress Hills were heterozygous for both ITS2 clusters, 38 of which belonged to Clade B. All snails sequenced or screened with clade specific primers from the Rocky Mountains belonged to ITS2 Cluster 2 (Table 3). All 41 individuals from the Rocky Mountains belonging to Clade B′ contained ITS2 Cluster 2 alleles.

**Table 2:** Number of individuals belonging to each COI clade and ITS2 cluster for 474 samples of *Oreohelix* snails. *Oreohelix subrudis* clades are represented by B, B′, and X. *Oreohelix cooperi* is represented by Co

ITS2 Cluster	COI Clade			
	B	B′	X	Co
1	56		128	
2	111	41	5	
1/2	38		6	
Co				89
Total	205	41	139	89

**Table 3:** Summary of genetic polymorphism statistics for ITS2 sequence data for *Oreohelix* spp. analyzed by site and region including number of individuals (n), alleles (A), number of variable sites (V sites), and allelic (h) and nucleotide (π) diversities. Site locations are described in Fig. 4. *Oreohelix subrudis* clades are represented by B, B′, and X. *Oreohelix cooperi* is represented by Co

Site	Cluster	n	A	V sites	h ± SD	π ± SD
CH01	Co	8	3	32	0.34 ± 0.14	0.0151 ± 0.0074
CH02	1/2	13	10	33	0.88 ± 0.03	0.0166 ± 0.0032
CH03	1/2	5	4	10	0.60 ± 0.13	0.0064 ± 0.0023
CH05	1/2	8	5	12	0.61 ± 0.13	0.0069 ± 0.0022
CH06	1/2	8	10	17	0.87 ± 0.08	0.0116 ± 0.0026
CH07	1/2	8	3	2	0.51 ± 0.13	0.0014 ± 0.0004
CH09	Co	5	5	70	0.67 ± 0.16	0.0410 ± 0.0147
CH10	Co	8	2	1	0.23 ± 0.13	0.0006 ± 0.0003
CH11	1/2	8	5	14	0.61 ± 0.13	0.0093 ± 0.0028
CH12	1	4	2	1	0.43 ± 0.17	0.0010 ± 0.0004
CH15	1	8	2	1	0.40 ± 0.11	0.0009 ± 0.0003
RM03	2	1	1	0	NA	NA
RM04	2	3	1	0	NA	NA
RM06	2	4	1	0	NA	NA
RM11	2	4	1	0	NA	NA
RM15	2	8	1	0	NA	NA
RM16	2	8	1	0	NA	NA
CH	1/2/Co	83	24	84	0.73 ± 0.03	0.0303 ± 0.0025
RM	2	28	1	0	NA	NA
Total	1/2/Co	114	23	82	0.75 ± 0.02	0.0259 ± 0.0021

### Morphological characteristics

Oreohelid snails collected from shrub-dominated slopes were *O. cooperi* (CH09 and CH10), measuring 8.5 ± 0.1 mm (95% CI) in maximum shell width. Average shell width of mitochondrial Clade B (sites CH03 and CH04) was almost double the size of *O. cooperi* at 15.0 ± 0.3 mm, while snails belonging to mitochondrial Clade X (sites CH07 and CH08) measured 15.4 ± 0.4 mm in width. An unpaired *t*-test showed that mean shell sizes of *O. cooperi* were significantly smaller than either of the two large-bodied snails (B and *O. cooperi*: t_118_ = 39.4; *p* < 0.0001; X and *O. cooperi*: t_118_ = 32.8; *p* < 0.0001), but the two large morphs where not significantly different in shell size from each other (t_118_ = 1.20; *p* = 0.23).

## Discussion

We obtained Oreohelid snails from 58 sites in two areas of southern Alberta and Saskatchewan. Two closely-related mitochondrial DNA clades were found in the Rocky Mountains, and three clades were found in Cypress Hills. Of the four COI clades identified (B, B′, X and Co), only *Oreohelix cooperi* (Co) was morphologically distinguishable [15, 18, 19]. The larger snails belong to the species complex *Oreohelix subrudis*, and are widespread throughout the Rocky Mountains and sky islands in Montana and Wyoming [12]. Two of the lineages sampled in Canada had highly restricted ranges: Clades X and B′. Clade X has only been found in Cypress Hills and is most closely related to Clade E in Wyoming described by [12] indicating that Clade X is either a glacial relict in Cypress Hills or colonized Cypress Hills from the periglacial zone immediately south of Cypress Hills and the Laurentide ice sheet. Clade B′ is restricted to the northern end of the sampled range in the Rocky Mountains and is closely related to Clade B. This proximity suggests that Clade B′ recently diverged from Clade B in the Rocky Mountains. The overall pattern of Oreohelid diversity in this region is one in which a set of narrow-range species (*O. cooperi*, Clade X and Clade B′) co-occur with at least one geographically widespread species (*O. subrudis*). This phylogeographic pattern is concordant with findings involving other species of Oreohelid snails studied on sky islands in the western United States [10] and with other land snails on isolated sky islands in South Africa [20] and India [21].

High levels of genetic divergence at the mitochondrial locus and cryptic species are commonly reported in studies of terrestrial snails [8–10, 22]. Thomaz et al. [23] attributes the high divergence to one of four possibilities: (i) relatively rapid mitochondrial divergence, (ii) prolonged isolation followed by secondary contact, (iii) selection pressure to maintain multiple mitochondrial clades, or (iv) colonization patterns leading to the co-occurrence of many divergent mitochondrial clades. The COI patterns observed in *Oreohelix* are best described by a combination of (ii) and (iv). Secondary contact following prolonged isolation (ii) best describes the presence of the three divergent mitochondrial lineages in Cypress Hills, particularly Clades B and X where nuclear ITS2 data show limited mixing. One possibility is that each of the clades of the *O. subrudis* species complex evolved in isolation on a sky island. Temperature and precipitation changes characterizing the Pleistocene glaciations allowed some of these clades to come into secondary contact. During the temperature and precipitation fluctuations in the early Holocene [24, 25], Cypress Hills and other sky islands such as the Black Hills or Bighorn Hills remained as relatively stable habitat for Oreohelids [26]. Secondary contact following prolonged isolation has been found in the dusky Arion slug (*Arion subfuscus* [27]). Pinceel et al. [27] reported highly divergent mitochondrial sequence data with low nuclear ITS1 divergence, which matches the pattern we found in our Oreohelid snails. Pinceel et al. [27] suggests that hybridization of nuclear loci occurred in the slugs, leading to the maintenance of multiple divergent mitochondrial lineages. Alternatively, multiple colonizations of divergent mitochondrial lineages (iv) could account for the current distribution of *O. cooperi* and *O. subrudis* in North America, including Cypress Hills. Under this model, an area is colonized either actively through a habitat corridor or through passive long-range dispersal [28–30]. The new population quickly expands in size, thereby reducing the likelihood of further colonizations. Both *O. cooperi* and COI Clade B are found in multiple, isolated populations and could have migrated north following the LGM. Colonization of multiple divergent mitochondrial clades explains the distribution of mitotypes in grove snails (*Cepea nemoralis*) across northwestern Europe [23] where populations are effectively arranged like stepping stones, and dispersal events between patches of suitable habitat are rare.

### ITS2 and cytonuclear discordance

ITS2 data revealed three genetic clusters; one in the Rocky Mountains, and three in Cypress Hills. One cluster was exclusive to *O. cooperi*. These results confirm those from our earlier study showing *O. cooperi* is genetically distinct from other sympatric Oreohelids and, within Canada, it is restricted to the Cypress Hills [18]. Snails in Cluster 1 were typical of *O. sp. X* and Cluster 2 was typical of *O. sp. B*, but the two divergent mitochondrial clades showed overlap at the ITS2 locus, particularly surrounding the contact zone in the Cypress Hills. There are two possibilities why Clades B and X exhibit mitochondrial and nuclear discordance: 1) incomplete lineage sorting or 2) introgression and hybridization [31]. While these are not mutually exclusive, hybridization and introgression better explain the ITS2 pattern observed in Cypress Hills. If incomplete lineage sorting was occurring, there would be no geographic pattern to the nuclear allele distribution in Cypress Hills [32]. However, there is a clear geographic pattern to both the mitochondrial COI and the nuclear ITS2. While both alleles are common in a small area surrounding the contact zone near Elkwater Lake, to the east of the contact zone near Highway 41 alleles belonging to Clade 1 become much more common.

### Glacial history

Oreohelids fossils in North America date back to the Cretaceous period [33], but the current distribution of Oreohelids in southern Alberta is a consequence of the Quaternary glacial history of the region. During the LGM, Cypress Hills was inaccessible to terrestrial snails and surrounded by ice and any populations of snails in Cypress Hills would have been isolated from other populations. As the ice sheets receded, the southern slopes were the first to become ice free and allow access to the hills, while the northern slopes retained ice for much longer [34]. Recent work on zonal reconstruction of vegetation from sites across North America suggests that 13,000 to 14,000 years ago, before the start of the late glacial warming period, Cypress Hills and the Sweet Grass Hills were the meeting point between the western Cordilleran forest and the eastern boreal forest in a thin zonal band adjacent to the Laurentide ice sheet [35]. The band of Cordilleran forest extended from west of Cypress Hills to the Rocky Mountains. While it did not extend far into the ice-free corridor of the Rocky Mountains until much later [36], the forest did remain adjacent to the Rocky Mountains in a narrow band following the Rocky Mountains south into Montana. As such, the Cordilleran forest was continuous with site MT1, the only location in the U.S. where Clade B has been found [12]. The band of boreal forest that extended from Cypress Hills is also thought to have been connected to the Black Hills, a site where *O. cooperi* is common. These bands of forest were transient and replaced by prairie within 2000 years [37]. While the most recent glacial maximum reached the farthest south, previous glacial maximums could have resulted in forests connecting to Cypress Hills as well, providing earlier corridors [38]. Although sky islands such as Cypress Hills and the Black Hills were once connected, the extent to which Oreohelids used these corridors is uncertain. The vegetation would have been capable of supporting Oreohelids, however, they also require the presence of other abiotic factors, such as calcareous deposits [15]. Limited fossil evidence exists of Oreohelids east of Montana during the most recent forest expansion [39, 40], so they were either present in low densities or absent.

### Glacial refugia and colonization

Cypress Hills may have acted as a glacial refugium for at least some Oreohelids during the LGM. Some studies suggest the Cypress Hills served as a refugia for species such as lodgepole pine (*Pinus contorta*) [41, 42]. Clade X is the only clade in Cypress Hills that is not present on other sky islands to the south or elsewhere in their range and is therefore likely a glacial relict. It is disjunct from its closest relative, Clade E, in northern Wyoming (WY2) over 500 km to the south. Further evidence that Clade X is a glacial relict is the pattern of the ITS2 network and distribution of Clades X and B (Fig. 3). Most individuals in Clade X contain ITS2 alleles from Cluster 1 and Cluster 1 is only found in the Cypress Hills.

Clade B is likely a post-glacial introduction to the Cypress Hills although the source of these colonists is not known. These individuals occupy a much smaller proportion of available habitat than Clade X, interestingly around the township, and Clade B is a relatively widespread lineage. Clade B is found at several sites in the Rocky Mountains (MT1, RM). The presence of Clade B′ in the Rocky Mountains indicates that Clade B was present in the Rocky Mountains long enough for Clade B′ to diverge. The sharing of ITS2 alleles between two mitochondrial groups, Clades B and X, in Cypress Hills indicates introgression between the two clades.

Clades B, C, D, E, and X from our study and [12] are relatively closely related. This species complex likely originated in Montana or Wyoming as most of the diversity occurs in that area, and Oreohelids have been present in Yellowstone Park area for more than 5 million years [33]. Temperature and precipitation fluctuations of the Pleistocene and early Holocene may have allowed colonization of the sky islands via habitat corridors followed by isolation in each sky island. Ice caps covered many of the sky islands that are currently occupied by Oreohelid land snails, but the distances between them are relatively small and sky islands were connected by suitable habitat for longer periods of time [12, 43]. Similar patterns have been found in glacial relict montane grasshoppers (*Melanoplus sp.*) in the same area [44, 45].

The restricted distribution of Clade B′ in the Rocky Mountains suggests more recent separation and divergence in situ from Clade B. The divergence between Clades B and B′ is much less than the divergence between any of the other subspecies of *O. subrudis* [12]. Clade B′ also has reduced ITS2 diversity. The Rocky Mountains foothills contained tundra-like vegetation throughout most of the Wisconsinan and an ice-free corridor throughout the last glacial maximum [36]. Trees did not colonize the area until approximately 8000 years ago, which is roughly concordant with pollen core data from Harris Lake ~ 25 km north of Cypress Hills [26, 36]. Despite extensive surveys, we have not found Oreohelids above 1600 m in the Rocky Mountains. At this elevation snow often remains year-round in southern Alberta and tundra-like, glacial relict vegetation is common.

The COI haplotypes found in *O. cooperi* from Cypress Hills are shared with snails from both the Judith Mountains and Black Hills. The original source of *O. cooperi* is likely somewhere within or near Wyoming, which contains *O. cooperi*, *O. pygmaea*, and Clade A.

### Contemporary patterns

The divide between Clades B and X in Cypress Hills is associated with Highway 41. Highways have been demonstrated to be substantial barriers to terrestrial snails [46]. Highway 41 was constructed in the early 1900’s and does not explain the presence of two distinct lineages on either side. The highway may currently reduce gene flow between Clades B and X, but the presence of the two clades in Cypress Hills predates the highway. The niches occupied by both Clades B and X are indistinguishable in Cypress Hills, and these snails have high population densities which may resist colonization by other taxa. Terrestrial snails are known for their ability to rapidly colonize or invade novel ecosystems due to their high reproductive capacity [47]. The spread of the colonizing snails would only stop at the edge of suitable habitat or an occupied niche [48, 49]. Even in highly vagile species such as pipits (*Anthus trivialis* and *A. spinoletta*) and buntings (*Emberiza citronella* and *E. hortulana*), long-term species segregation is possible through the combination of factors including habitat differences and interspecific competition [50].

While hybridization between the two mitochondrial clades is evident, it is limited to a narrow area and the introgression of ITS2 alleles is asymmetrical. Many more Clade B snails share ITS2 alleles with Clade X than the reverse. Oreohelids are simultaneous hermaphrodites, yet the pattern is similar to male-biased dispersal in dioecious organisms [31] suggesting unequal movement between male and female gametes in the contact zone. It could be that these snails seek out mates, but return to their usual resting place to give birth. This would mimic male-biased dispersal, and reduce inbreeding. Another possibility is asymmetrical mating, where snails may behave as males for some mates but females for others. Asymmetrical mating has been demonstrated in land snails including Oreohelids [51, 52] and has been observed in Oreohelids from Cypress Hills (Z. Dempsey, personal observations).

## Conclusions

Using molecular techniques, three mitochondrial clades of Oreohelids have been identified in Cypress Hills, and two in the Rocky Mountains. Two of these lineages have not been found previously despite extensive genetic surveys in the U.S. The small morph of Oreohelid snail in Cypress Hills, identified as *O. cooperi*, has been demonstrated to be genetically distinct from large-bodied snails of Cypress Hills and the Rocky Mountains. We propose that some lineages of Oreohelids in Cypress Hills and the Rocky Mountains are glacial relicts and others are now restricted to niches that were much more widespread during the glacial maximum.

## Methods

### Study areas and sampling

Cypress Hills Interprovincial Park (CHP) is situated on the southern Canadian *plains* (49^o^ 30′N, 110^o^W) in the provinces of Alberta and Saskatchewan covering an area of approximately 2590 km^2^ [17]. The hills (maximum elevation of 1420 m a.s.l.) comprise a plateau of pre-glacial bedrock that rises 430 m above the surrounding prairie [53]. The hills were surrounded by glaciers twice during the Quaternary period, but the plateau above approximately 1350 m remained unglaciated [54]. Highland forest and grassland communities found in CHP are most similar to those of the Rocky Mountains located 300 km to the west and to the aspen parkland regions characteristic of the central areas of Canada’s three prairie provinces [17]). Forested areas comprise approximately 20% of CHP, with the remainder comprised of fescue grassland and wetland habitats [17]. *Pinus contorta* (lodgepole pine), *Populus tremuloides* (trembling aspen), *Picea glauca* (white spruce), and *Populus balsamifera* (balsam poplar) are the dominant tree species [17]. Grassland communities are dominated by *Festuca campestris* (rough fescue) and other grasses that characterize the mixed-grass natural sub-region [55]. Other habitat characteristics that are relevant to the occurrence of Oreohelid snails in CHP are described in [18, 56].

**Fig. 4:**
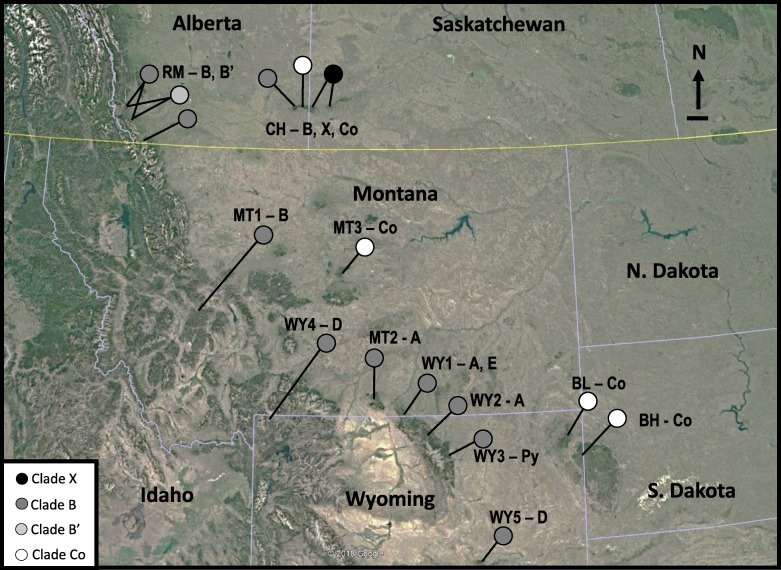
Satellite image of all *Oreohelix* sampling sites including [10]. Sites are coded based on COI clade present. Samples include *O. cooperi* (Co), *O. pygmaea* (Py), and *O. subrudis* (Clades B-E). The scale bar represents 20 km. Map Data: Google Earth, CNES/Airbus, Landsat/Copernicus, Maxar Technologies (2018). *Oreohelix subrudis* clades are represented by B, B′, and X. *Oreohelix cooperi* is represented by Co

The forested slopes of CHP provide ideal habitat for Oreohelid snails [15, 17, 18]. Similarly, the Rocky Mountains located to the west contain a patchwork of suitable conditions for Oreohelid snails that are interconnected with habitat corridors [57]. Contained in the Rocky Mountains of southwestern Alberta is Waterton Lakes National Park (WLNP). This park comprises a wide range of montane habitats including mountains, prairie, lakes, and wetlands. Vegetation consist primarily of fescue grasslands and deciduous forests including aspen forests at lower elevations and a transition to alpine meadows and coniferous forests at higher elevations [57]. Sites within the Castle Wildlands Area and Crowsnest Pass have similar habitats to those in Waterton Lakes National Park (Fig. 4).

**Fig. 5:**
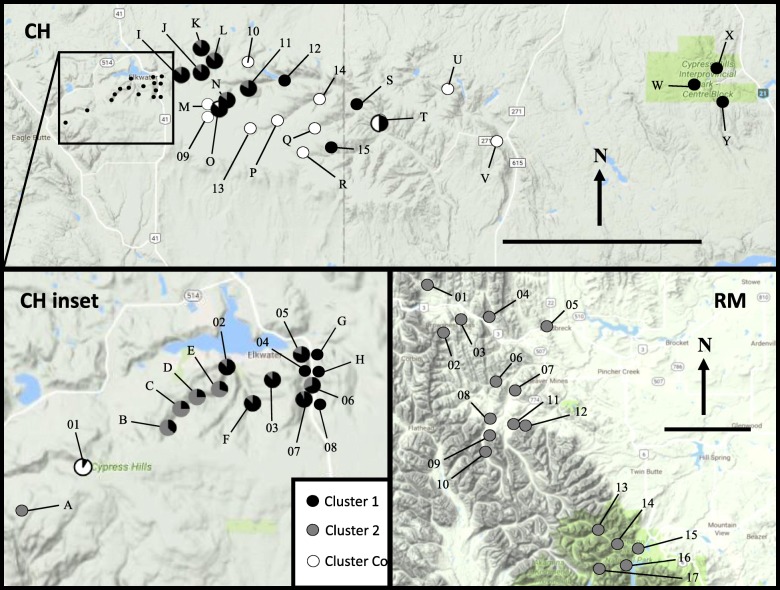
Relief image of *Oreohelix* ITS2 sequencing (numbers) and screening (letters) results in Cypress Hills (CH) and the Rocky Mountains (RM). Refer to Table 3 for sample sizes and ITS2 diversity estimates. The scale bar in each inset represents 20 km. Map Data: Google Earth, CNES/Airbus, Landsat/Copernicus, Maxar Technologies (2018). *Oreohelix subrudis* clades are represented by B, B′, and X. *Oreohelix cooperi* is represented by Co

Individual snails (*n* = 474) were obtained from 58 sites in southern Alberta and Saskatchewan throughout the snow-free months of 2013–2016 (Figs. 2 and 5). During visits to each site, plots approximately 10 m^2^ were demarcated with flagging tape. A maximum of 30 Oreohelid snails were collected as they were encountered and preserved in 90% ethanol following methods in [56]. All sample sites were searched by two people for at least 30 min. Snails were stored in ethanol at − 80 °C. Data on slope, aspect, and elevation were recorded for each site.

Assessments of shell characteristics namely maximum shell width was determined for 30 adult snails per site. For morphological assessments, snails from six sites (CH03, CH04, CH07, CH08, CH09, and CH10) were selected based on preliminary genetic data, representing each of the three main mtDNA clades [18].

### DNA extraction, amplification, and sequencing

Approximately 2 mg of foot tissue from each snail was used for a modified chelex DNA extraction following [18, 58]. Extracted DNA was amplified with COI barcoding primers LCO1490 and HCO2198 [59] and ITS2 primers LSU1F2990 and ITS4R3908 [60]. A 585 bp fragment of the COI locus was amplified under the following conditions: 1 cycle of 94 °C for 2 min, 50 °C for 45 s, and 72 °C for 1 min; 37 cycles of 94 °C for 30 s, 50 °C for 45 s, and 72 °C for 1 min, with a final cycle at 72 °C for 5 min. The PCR mix contained: 1 Unit Flexi DNA polymerase, 1x Flexi buffer, 0.2 mM dNTP, 0.4 μM of each primer, and 3 mM MgCl_2_. The ITS2 amplification of a 472 bp region followed the same protocol, with the exception of a 48 °C annealing temperature and 1 mM MgCl_2_. Amplified DNA was sent to McGill University for sequencing.

### Sequencing data analyses

Sequences were aligned, trimmed and overall sequence divergence at each locus was calculated in MEGA 6.06 [61]. DnaSP 5.1 was used to calculate haplotype (h) or allelic (A) and nucleotide (π) diversities for both loci [62]. Maximum likelihood models of phylogenetic tree construction were ranked based on Akaike Information Criterion corrected (AICc) for finite sample sizes using PAUP* 4.0a151 [63]. The model with the lowest AICc score was used to construct a phylogenetic tree that was validated with 1000 bootstrap replicates with 50% consensus. TreeGraph 2.9.2 [64] was used to create the final tree. After sequences for heterozygotes were converted to individual alleles using PHASE in DnaSP, ITS2 sequence data were used to construct a minimum spanning network with 300 iterations and 0 epsilon in PopART 1.7 [65]. A maximum likelihood tree using Jukes-Cantor model was also used for comparison.

### Screening primers

Both COI and ITS2 data showed fixed differences between clades such that screening primers were developed targeting fixed nucleotide differences to type the remaining samples. The forward or L strand primers were designed to target clade specific sequences and, in combination with the common reverse or H strand primer, to produce different sized bands. The COI locus used a common primer HCOIcommO (5′ – TAA ACT TCA GGG TGA CCA AAA – 3′) with three clade specific primers in a multiplex PCR: LCOIspecOc635 (5′ – TGC TCT TTC ACT TTT AAT TCG AC – 3′) only amplified *Oreohelix cooperi*, LCOIspecOs563 (5′ – ATT GTT ACA GCC TAT GCC – 3′) only amplified *Oreohelix sp. B*, and LCOIspecOx217 (5′ – GTG CCC CAG GAA TAA ATT TG – 3′) only amplified *Oreohelix sp. X*. The COI screening primer used a 48 °C annealing temperature and 1 mM MgCl_2_ producing bands at 635 bp, 563 bp and 217 bp for *O. cooperi*, *sp. B* and *sp. X*, respectively. The ITS2 locus used a common reverse primer ITS4R3908 with three different forward primers in a multiplex PCR: ITS2FspecOc256 (5′ – CCG TGG TCT TAA GTT CAA A – 3′) amplified *O. cooperi*, ITS2FspecOs112 (5′ – TTA ACG AAA AGT GGA TGC T – 3′) preferentially amplified *O. sp. B*, and ITS2specOx356 (5′ – CTG CTG TGC TCT AGC ATT TAT – 3′) preferentially amplified *O. sp. X*. The ITS2 screening PCR had a 54 °C annealing temperature and 1.5 mM MgCl_2_. Amplified DNA was visualized and scored on 3% agarose gel stained with ethidium bromide. ITS2 PCR products were 256 bp, 112 and 356 bp fragments for *O. cooperi*, *O. sp. B,* and *O. sp. X*, respectively. Multiple samples from each of the COI and ITS2 clades were sequenced and screened with the clade specific primers to ensure accurate screening.

## Supplementary information

**Additional file 1.** General summary information about each sample site.

**Additional file 2.** Information about each sample, taken during time of sampling. Includes old sample names.

**Additional file 3.** File used to create PAUP* phylogenetic tree containing data from this study as well as Weaver et al. [12]. (FAS 213 kb)

**Additional file 4.** Input file for COI GenBank submission. Sequences have been trimmed to all have the same reading frame. (FAS 157 kb)

**Additional file 5.** General input file for COI, used to calculate measures such as haplotype diversity, nucleotide diversity, etc. (FAS 161 kb)

**Additional file 6.** Input file for ITS2 GenBank submission. Sequences have been trimmed to all have the same reading frame. (FAS 108 kb)

**Additional file 7.** General input file for ITS2, used to calculate measures such as haplotype diversity, nucleotide diversity, etc. (FAS 131 kb)

**Additional file 8.** Input file for Popart, used to create haplotype network.

## Data Availability

Sequences generated are available under GenBank accession numbers MN695417-MN695891. The datasets supporting the conclusions of this article are available through GenBank or as supplementary files on the journal’s website. See the Supplementary File Guide for further information on each supplemental file. Supplemental files include: Additional file 1, Additional file 2, Additional file 3, Additional file 4, Additional file 5, Additional file 6, Additional file 7, and the Additional file 8.

## Abbreviations

°C: Degrees Celsius
CHP: Cypress Hills Park
COI: Cytochrome oxidase I
DNA: Deoxyribonucleic acid
ITS2: Internal transcribed spacer 2
mtDNA: Mitochondrial DNA
PCR: Polymerase chain reaction
