# Supplementary material for: Living on the edge: comparative phylogeography and phylogenetics of Oreohelix land snails at their range edge in Western Canada
Source: BMC Evol Biol. 2020 Jan 6;20:3. doi: 10.1186/s12862-019-1566-1 (PMC6945528; doi:10.1186/s12862-019-1566-1)
Supplement: Supplementary file 1 — Additional file 1. General summary information about each sample site. [file 12862_2019_1566_MOESM1_ESM.pdf]

Supplemental information

Location information and summary of all samples screened and sequenced as part of this study. Sites marked with * appeared in Dempsey et al. (2019). *Oreohelix subrudis* clades are represented by B, B', and X. *Oreohelix cooperi* is represented by Co. GenBank accession numbers refer to numbers for each haplotype. Where individuals share the same haplotype, they have the same sample accession number in the following table. The sequenced Popset includes accession numbers MN695417 - MN695891.

Site	n	mtClade	ITS cluster	Lat	Long	COI accession numbers	ITS2 accession numbers
**CH01***	11	B/Co	2/Co	49.6322	-110.3614	MN695469, 84	MN695664, 730
**CH02**	20	B	1/2	49.6570	-110.2954	MN695469	MN695820, 664
**CH03**	8	B	1/2	49.6581	-110.2808	MN695469	MN695820, 664
**CH04**	10	B	1	49.6591	-110.2614	MN695469	MN695820
**CH05**	8	B	1/2	49.6648	-110.2636	MN695469	MN695820, 664
**CH06**	8	X/B	1/2	49.6560	-110.2611	MN695469, 530	MN695820, 664
**CH07**	8	X	1/2	49.6503	-110.2614	MN695530	MN695820, 664
**CH08**	12	X	1	49.6500	-110.2571	MN695530	MN695820
**CH09***	8	Co	Co	49.6288	-110.1852	MN695484	MN695730
**CH10***	8	Co	Co	49.6722	-110.1470	MN695484	MN695730
**CH11**	12	X	1/2	49.6610	-110.1175	MN695530	MN695820, 664
**CH12**	10	X	1	49.6638	-110.0723	MN695530	MN695820
**CH13***	8	Co	Co	49.6179	-110.0936	MN695484	MN695730
**CH14***	8	Co	Co	49.6421	-110.0334	MN695484	MN695730
**CH15**	10	X	1	49.5998	-110.0239	MN695530	MN695820
**CHA**	8	B	2	49.6176	-110.3892	MN695469	MN695664
**CHB**	8	B	1/2	49.6401	-110.3224	MN695469	MN695820, 664
**CHC**	8	B	1/2	49.6449	-110.3179	MN695469	MN695820, 664
**CHD**	8	B	1/2	49.6506	-110.3064	MN695469	MN695820, 664
**CHE**	8	B	1/2	49.6511	-110.2986	MN695469	MN695820, 664
**CHF**	8	B	1/2	49.6509	-110.2857	MN695469	MN695820, 664
**CHG**	8	B	1	49.6643	-110.2604	MN695469	MN695820
**CHH**	8	B	1	49.6578	-110.2599	MN695469	MN695820
**CHI**	8	X	1	49.6704	-110.2399	MN695530	MN695820
**CHJ**	8	X	1/2	49.6831	-110.1948	MN695530	MN695820, 664
**CHK**	8	X	1/2	49.6878	-110.1980	MN695530	MN695820, 664
**CHL**	8	X	1/2	49.6784	-110.1852	MN695530	MN695820, 664
**CHM**	8	Co	Co	49.6377	-110.2067	MN695484	MN695730
**CHN**	8	Co/X	1/2/Co	49.6384	-110.1658	MN695484, 530	730, 820, 664
**CHO**	8	Co/X	1/Co	49.6348	-110.1639	MN695484, 530	MN695730, 820
**CHP***	8	Co	Co	49.6129	-110.1494	MN695484	MN695730
**CHQ***	8	Co	Co	49.6110	-110.0585	MN695484	MN695730
**CHR***	8	Co	Co	49.5930	-110.0862	MN695484	MN695730
**CHS***	8	X/Co	1/Co	49.6338	-109.9852	MN695484, 530	MN695730, 820
**CHT**	8	X	1	49.6468	-109.9991	MN695530	MN695820
**CHU***	8	Co	Co	49.6462	-109.8468	MN695484	MN695730
**CHV***	1	Co	Co	49.6027	-109.7958	MN695484	MN695730
**CHW**	8	X	1	49.6596	-109.5166	MN695530	MN695820
**CHX**	8	X	1	49.6688	-109.5001	MN695530	MN695820
**CHY**	8	X	1	49.6515	-109.4969	MN695530	MN695820
**RM01**	8	B	2	49.6789	-114.6098	MN695469	MN695664

**RM02**	8	B/B’	2	49.5973	-114.4978	MN695469, 38	MN695664
**RM03**	8	B	2	49.6040	-114.4981	MN695469	MN695664
**RM04**	8	B/B’	2	49.6015	-114.3970	MN695469, 38	MN695664
**RM05**	8	B	2	49.5833	-114.2071	MN695469	MN695664
**RM06**	8	B’	2	49.4525	-114.4097	MN695438	MN695664
**RM07**	8	B/B’	2	49.4445	-114.3239	MN695469, 38	MN695664
**RM08**	8	B’	2	49.3807	-114.3666	MN695438	MN695664
**RM09**	8	B’	2	49.3494	-114.4063	MN695438	MN695664
**RM10**	8	B’	2	49.3584	-114.4065	MN695438	MN695664
**RM11**	8	B	2	49.3666	-114.3042	MN695469	MN695664
**RM12**	8	B	2	49.3633	-114.2939	MN695469	MN695664
**RM13**	8	B	2	49.1286	-114.0254	MN695469	MN695664
**RM14**	4	B	2	49.0970	-113.9660	MN695469	MN695664
**RM15**	8	B	2	49.0770	-113.8814	MN695469	MN695664
**RM16**	8	B	2	49.0370	-113.9126	MN695469	MN695664
**RM17**	8	B	2	49.0662	-113.9981	MN695469	MN695664
